# Regeneration of a full-thickness defect of rotator cuff tendon with freshly thawed umbilical cord-derived mesenchymal stem cells in a rat model

**DOI:** 10.1186/s13287-020-01906-1

**Published:** 2020-09-07

**Authors:** Ji-Hye Yea, Jin-Kyung Park, In Ja Kim, Gayoung Sym, Tae-Soo Bae, Chris Hyunchul Jo

**Affiliations:** 1 Department of Translational Medicine, Seoul National University College of Medicine, 103 Daehak-ro, Jongno-gu, Seoul, 03080 Korea; 2 Department of Orthopedic Surgery, SMG-SNU Boramae Medical Center, Seoul National University College of Medicine, 20 Boramae-ro 5-gil, Dongjak-gu, Seoul, 07061 Korea; 3 Department of Biomedical Engineering, Collage of Science and Engineering, Jungwon University, 85, Munmu-ro, Goesan-eup, Goesan-gun, Chungcheongbuk-do 367-805 Korea

**Keywords:** Rotator cuff, Tendon regeneration, Mesenchymal stem cells, Cryopreservation

## Abstract

**Background:**

It is difficult to immediately use mesenchymal stem cells (MSCs) for the patient with rotator cuff disease because isolation and culture time are required. Thus, the MSCs would be prepared in advanced in cryopreserved condition for an “off-the-shelf” usage in clinic. This study investigated the efficacy of freshly thawed MSCs on the regeneration of a full-thickness tendon defect (FTD) of rotator cuff tendon in a rat model.

**Methods:**

We evaluated morphology, viability, and proliferation of cultured umbilical cord-derived MSCs (C-UC MSCs) and freshly thawed umbilical cord-derived MSCs (T-UC MSCs) at passage 10 in vitro. In animal experiments, we created a FTD in the supraspinatus of rats and injected the injured tendon with saline, cryopreserved agent (CPA; control), C-UC MSCs, and T-UC MSCs, respectively. Two and 4 weeks later, macroscopic, histological, biomechanical, and cell trafficking were evaluated. *T* test and ANOVA were used with SPSS. Differences with *p* < .05 were considered statistically significant.

**Results:**

T-UC MSCs had fibroblast-like morphology and showed greater than 97% viability and stable proliferation comparable to the C-UC MSCs at passage 10. In animal experiments, compared with the control group, the macroscopic appearance of the T-UC MSCs was more recovered at 2 and 4 weeks such as inflammation, defect size, neighboring tendon, swelling/redness, the connecting surrounding tissue and slidability. Histologically, the nuclear aspect ratio, orientation angle of fibroblasts, collagen organization, and fiber coherence were improved by 33.33%, 42.75%, 1.86-fold, and 1.99-fold at 4 weeks, and GAG-rich area decreased by 88.13% and 94.70% at 2 and 4 weeks respectively. Further, the T-UC MSCs showed enhanced ultimate failure load by 1.55- and 1.25-fold compared with the control group at both 2 and 4 weeks. All the improved values of T-UC MSCs were comparable to those of C-UC MSCs. Moreover, T-UC MSCs remained 8.77% at 4 weeks after injury, and there was no significant difference between C-UC MSCs and T-UC MSCs.

**Conclusions:**

The morphology, viability, and proliferation of T-UC MSCs were comparable to those of C-UC MSCs. Treatment with T-UC MSCs could induce tendon regeneration of FTD at the macroscopic, histological, and biomechanical levels comparable to treatment with C-UC MSCs.

## Background

Rotator cuff disease is a major cause of shoulder pain, and approximately 300,000 operations are performed each year in the USA [1, 2]. After conservative treatments such as rest, non-steroidal anti-inflammatory drugs, physical therapy, and various kinds of injections [3], at least 45% patients suffer from persistent symptoms even after 12 months [4]. These symptoms are attributed to the low healing potential of the rotator cuff tendon due to the avascular and acellular structure, and the tenocytes no longer participate in the regeneration of tendon after injury [5]. Thus, spontaneous healing of tendon disease is a difficult proposition.

Recently, mesenchymal stem cells (MSCs) have emerged as a promising candidate for fundamental tissue regeneration of rotator cuff tendon [6]. Bone marrow-derived MSCs (BM MSCs) and adipose tissue-derived MSCs (AD MSCs) improve collagen organization and collagen fiber coherence and enhance the tensile strength of tendon in a rat model of rotator cuff injury [7, 8]. However, the use of these MSCs entails invasive harvesting techniques [9], low collection efficiency [10], decreased ability with age, and donor morbidities [11]. Especially, heterotopic bone formation induced by BM MSCs is a crucial risk factor limiting clinical application. Umbilical cord-derived MSCs (UC MSCs) represent an alternative cell source. UC MSCs isolated from the umbilical cord, which is a medical waste following delivery, could be obtained non-invasively and at relatively low cost [10]. It has higher proliferative and self-renewal potential than other adult MSCs [12]. In regenerative medicine, it is reported that UC MSCs can be used to recover tissue structure in a mouse model of ischemic injury [13] and a C57BL6 mouse model of wound injury [14]. Thus, UC MSCs could be used to potentially recover the tendon tissue of rotator cuff.

For clinical application, an “off-the-shelf” usage with allogeneic MSCs is more promising than usage after isolation and culture in each case, or usage with rescue culture for several days after thawing for allogeneic MSCs. However, MSCs might be subjected to substantial physiological changes such as cell growth, phenotype, differentiation, viability, and safety profile in vivo during the freshly thawing procedure [15, 16]. The results of applying freshly thawed MSCs to animal models vary with the study. The freshly thawed MSCs are impacted by factors associated with post-infusion biodistribution as they bind poorly with fibronectin, human endothelial cells, and cytoskeletal F-actin compared with cultured MSCs. Freshly thawed MSCs were undetectable in the lung tissues of C57BM/B6 mice whereas the cultured MSCs could be detected for up to 24 h [17]. In contrast, another study reported that the freshly thawed MSCs had a healing effect on allergic airway inflammation in a mouse model comparable to that of cultured MSCs [18]. These conflicting results suggest a potential risk associated with the clinical use of freshly thawed MSCs. However, the healing potential of MSCs is disease-specific and affected by factors such as tissue origin, donor variation, culture time, supplements, and other aspects associated with delivery [16]. Thus, the efficacy of specific cells under specific disease conditions remains to be investigated. Until now, although some studies investigated tissue healing using UC MSCs, the role of freshly thawed UC MSCs (T-UC MSCs) in tendon recovery for clinical use compared with continuously cultured UC MSCs (C-UC MSCs) has never been reported.

Therefore, this study investigated the efficacy of T-UC MSCs in a rat model of full-thickness tendon defect (FTD) of the supraspinatus tendon. We hypothesized that T-UC MSCs have comparable morphology, viability, and proliferation to those of C-UC MSCs. The T-UC MSCs could induce regeneration of tendon in terms of macroscopic, histological, and biomechanical characteristics comparable to C-UC MSCs.

## Methods

### UC MSCs isolation and culture

This study was approved by the Seoul Metropolitan Government Seoul National University Boramae Medical Center Institutional Review Board (IRB No. 16-2015-115). Informed consent was obtained from all patients before performing the study. Human umbilical cords were obtained from full-term birth via cesarean section. Isolated umbilical cords were washed 2 to 3 times with Dulbecco’s phosphate-buffered saline (DPBS; Welgene, Daegu, Korea) to remove blood products, and the length and weight were measured, followed by sectioning into minimal cube explants, each measuring 2–4 mm, using surgical scissors. The cube explants (1 g) were aligned at regular intervals in 15-cm culture dishes and allowed to firmly attach to the bottom of the dish for 60 min in a 5% CO_2_ incubator with humidified air at 37 °C. The culture medium consisting of low-glucose Dulbecco’s modified Eagle’s medium (LG-DMEM; Hyclone, Logan, USA) supplemented with 10% fetal bovine serum (FBS; Hyclone) and antibiotic-antimycotic solution (100 U/mL penicillin, 100 μg/mL streptomycin, and 0.25 μg/mL amphotericin B; Welgene) was gently poured into the dishes. The medium was replaced twice a week. Non-adherent cells were removed by changing the medium. When cells reached 80% confluency, they were detached by incubation for 3 min with trypsin-ethylendiaminetetracetic acid (EDTA) (0.05% trypsin, 0.53 mM EDTA; Welgene). The tissues were removed through a 100-μm cell strainer (SPL Life Sciences, Pocheon, Korea), and the cells were centrifuged at 500*g* for 5 min at 20 °C and then replated at a density of 3 × 10^3^ cells/cm^2^. The culture medium was changed every 2–3 days and continuously cultivated. The C-UC MSCs were continuously cultured up to passage 10 before use in experiments and characterized morphologically, in addition to the determination of growth kinetics, CFU-F, flow-cytometric, and trilineage differentiation of cells as reported previously [19]. The T-UC MSCs were cryopreserved with a cryoprotective agent (CPA) (including 10% DMSO; ZENOAQ RESOURCE, Fukushima, Japan). Cryovials were stored at − 80 °C in a deep-freezer and transferred to − 196 °C liquid nitrogen tank for preservation up to 1 month. For use in experiments, T-UC MSCs were thawed immediately in the 37 °C water bath within 1 min. The T-UC MSCs were also used at the same passage as C-UC MSCs for each experiment.

### Characterization of C- and T-UC MSCs

When C- and T-UC MSCs reached 80% confluence, cell morphologies were observed under a microscope (CKX53 Olympus culture microscope; Olympus, Tokyo, Japan).

Cell viability of C- and T-UC MSCs was determined by the trypan blue exclusion method and water-soluble tetrazolium salt (WST) assay. Cells were stained with trypan blue (0.4%) solution and the live and dead cells were counted using the hemocytometer. The percentages of cell viability of C- and T-UC MSCs were calculated using the following formula: total number of live cells/total number of cells × 100 (%). For WST assay, cell suspensions were plated at a density of 1 × 10^4^ cells/well on a 96-well plate and incubated for 0, 2, 6, 24, and 48 h in the presence of 5% CO_2_ with humidified air at 37 °C. At the respective time points, 10 μL WST solution (WST assay kit; EZ3000, DAEILLAB SERVICE CO. Ltd.) was added to each well for 2 h in a 5% CO_2_ incubator with humidified air at 37 °C. After the 2 h incubation, optical density (OD) was measured at 450 nm using a microplate spectrophotometer (Power Wave XS; Bio-Tek Instruments, Winooski, VT, USA).

Proliferative capacities of C- and T-UC MSCs were evaluated by calculating the cumulative population-doubling level (CPDL) and population-doubling time (PDT). Cells were plated at a density of 3 × 10^3^ cells/cm^2^ on a 6-well plate in triplicate. Cells were harvested every 4 days up to 20 days. PDT of cells was calculated using the following formula: PD = Log (*N*_*f*_/*N*_*i*_)/Log2, PDT = CT/PD, where CT denotes culture time, and *N*_*f*_ and *N*_*i*_ refer to the final and initial number of cell, respectively [20].

### In vivo study design

Animal procedures were conducted in accordance with the protocol approved by the Seoul Metropolitan Government Seoul National University Boramae Medical Center Institutional Animal Care and Use Committee (IACUC_2020_0004). One hundred twenty adult male Sprague-Dawley rats (12 weeks old, 340~360 g) were divided into one of the four groups and treated accordingly: (1) saline group, (2) CPA group, (3) C-UC MSC group, and (4) T-UC MSC group. Rats from each group were sacrificed immediately after surgery, 2 and 4 weeks after surgery. The supraspinatus tendon (SST) was harvested and used for macroscopic and histological evaluation (*n* = 4), biomechanical evaluation (*n* = 8), and cell trafficking (*n* = 4). Workflow was schematized in an Additional file 1.

### Surgical procedures

Anesthesia was induced using zoletil and rompun (30 mg/kg + 10 mg/kg). The left shoulder was operated in all cases. A 20cm skin incision was made directly over the anterolateral border of the acromion. After the SST was exposed by detaching trapezius and deltoid muscle from the acromion, a round FTD with a diameter of 2 mm in the middle of the SST was created 1 mm from the insertion using a Biopsy Punch (BP-20F, Kai Medical Europe GmbH, Bremen, Germany). This defect size was approximately 50% of the tendon width, correlating with a large but not a massive tear according to the method previously described [21]. Ten microliters of saline, CPA, C-UC MSCs (1 × 10^6^ cells in saline), and T-UC MSCs (1 × 10^6^ cells in CPA) were intratendinously injected adjacent to both sides of the defect in two divided doses using a 30-G needle. After injection, the deltoid and trapezius muscles were sutured with a 4–0 Vicryl suture (W9074, Ethicon, Cincinnati, OH, USA), and the skin was also sutured with a black silk (SK439, AILee, Busa, Korea). After surgery, animals were allowed free cage activity.

### Macroscopic evaluation

At 2 and 4 weeks after injection, the rats were sacrificed in a carbon dioxide chamber. The SST of the rats was harvested along with the humerus head without removing the muscle. For macroscopic evaluation, we used a modified semi-quantitative system described by Stoll et al. [22] (see Additional file 2). The 12 parameters in the system were tendon rupture, inflammation, tendon surface, neighboring tendon, level of the defect, defect size, swelling/redness of tendon, connection surrounding tissue and slidability, tendon thickness, color of tendon, single strain of muscle, and transition of the construct to the surrounding healthy tissue. Each parameter varied from 0 or 1 except for swelling/redness score (0 to 2) and tendon thickness score (0 to 3). Therefore, the total macroscopic score varied between 0 (normal tendon) and 15 (most severe injury).

### Histological evaluation

After the macroscopic evaluation, the harvested tissues were immediately fixed in 4% (w/v) paraformaldehyde (PFA; Merck, Darmstadt, Germany) for 24 h, followed by decalcification in 10% EDTA (Sigma-Aldrich, St Louis, MO, USA) for 2 days. After decalcification, the tissues were dehydrated through an increasing series of ethanol gradient, defatted in chloroform, and embedded in paraffin blocks. The tissue was carefully trimmed to the appropriate middle site of tendon and cut into 4-μm-thick serial sections.

A randomly selected slide was stained with hematoxylin and eosin (H&E) and analyzed by light microscopy (U-TVO 63XC; Olympus Corp., Tokyo, Japan). For the evaluation of tendinopathy, each slide was evaluated using the semi-quantitative grading scale as previously described [23]. The 7 parameters of the system include fiber structure, fiber arrangement, rounding of the nuclei, variations in cellularity, vascularity, stainability, and hyalinization. Each parameter in the grading scales varies from 0 to 3. The total degeneration score for a given slide varied between 0 (normal tendon) and 21 (most severely degenerated).

For the evaluation of inflammation, infiltration of inflammatory cells was evaluated using a 0–3 grading scale: 0 (normal), 1(slightly abnormal), 2 (moderately abnormal), and 3 (maximally abnormal) [24].

In normal tendon, the few fibroblasts with flattened nuclei are typically aligned parallel to the tensile axis. After injury, the morphometric changes of fibroblast nuclei were evaluated as previously described by Fernandez-Sarmiento using H&E stained slides [25]. Fibroblast density (number of nuclei per mm^2^), nuclear aspect ratio (the ratio of the minor diameter to the maximal diameter), and nuclear orientation angle (between the major axis of the nuclear angle and the axis of collagen fibers) were evaluated. Five regions of interest (ROI) were measured and the average was used finally.

We also evaluated the occurrence of heterotopic ossification when separated, clustered, and bar-shaped foci were found in the whole tendon structure [26].

Slides were also stained with picrosirius red (PSR) for analysis of collagen fiber organization and coherency using circularly polarized light microscopy at × 200 magnification. Collagen organization was measured as intense white areas of brightly diffracted light on gray scale (black, 0; white, 255) using ImageJ software with installed NII plugin (National Institutes of Health, MD, USA). Higher gray scale indicated more organized and mature collagen [27]. The coherence of the collagen fibers is a measure of the extent of fiber alignment in the major axis of alignment. The coherence was quantified using the Orientation J plug-in for ImageJ and then multiplied by 100 to obtain the final coherence value [7]. Five ROIs were measured and the mean value was used. Moreover, slides were stained with Masson’s trichrome to evaluate collagen deposit, and immunohistochemistry (IHC) was performed to assess type I collagen formation after injury, using rabbit anti-type 1 collagen (1:300 dilution, Abcam; ab34710). Detailed procedures are described in Additional file 3.

For evaluation of cartilage formation, slides stained with alcian blue were used and observed via light microscopy at × 40 magnification. The glycosaminoglycan (GAG)-rich area was measured using ImageJ [11, 28].

### Biomechanical evaluation

For biomechanical testing, we harvested supraspinatus tendon with humerus head and carefully removed the muscles to leave only the tendon. The harvested tissues were wrapped in saline-soaked gauze and kept at − 80 °C. Before testing, the tissues were thawed with saline wet gauze at room temperature for 24 h, and the tissues were kept moist with saline during all tests. The distal part of the humerus bone was vertically embedded in an aluminum tube full of polymethylmethacrylate (PMMA) in the custom-designed lower jig of a testing system. The proximal end of the tendon was compressed with sandpaper, gauge, and rubber to prevent slippage and to reduce damage of specimens. The complex was clamped vertically in the custom-designed upper jig. Testing was performed with shoulders at 90° of abduction with a material testing system (H5K5, Tinus Olsen, England, UK) [29, 30]. All specimens were loaded to failure in tension at a constant rate of 0.1 mm/s. Slippage of the tendon was inspected visually. The cross-sectional area of supraspinatus tendon was measured at the center of defect region and was calculated with the formula area = abπ/4. From the load-displacement curve recorded during tests, the ultimate failure load, the stiffness, and ultimate stress were calculated [28, 31].

### UC MSCs trafficking

UC MSCs were labeled with red fluorescent PKH26 (Sigma-Aldrich, St Louis, MO, USA) according to the manufacturer’s protocol [32]. Detailed procedures are described in Additional file 4. After labeling, the cells were counted by hemocytometer and were confirmed by fluorescence microscope (Leica DMI 4000B, Leica, Wetzlar, Germany). After injection, the tissues were harvested immediately after injection and at 2 and 4 weeks after injection and used for evaluation (see Additional file 4). Five fields were randomly selected in the slide and high-powered images (× 400) were obtained by fluorescence microscopy. The PKH26 positive cells coincident with 4′,6-diamidino-2-phenylindole (DAPI) were counted per area and the mean number was recorded by ImageJ [33].

### Statistical analysis

All data are shown as mean ± standard deviation. A *T* test was used to determine significance between the means of two groups in vitro, and animal experimental data were analyzed with one-way analysis of variance (ANOVA) with post hoc analysis using Bonferroni multiple comparison test. All statistical analyses were performed with SPSS software version 23 (IBM). Differences of *p* < 0.05 were considered statistically significant.

## Results

### Characterization of C- and T-UC MSCs

**Fig. 1:**
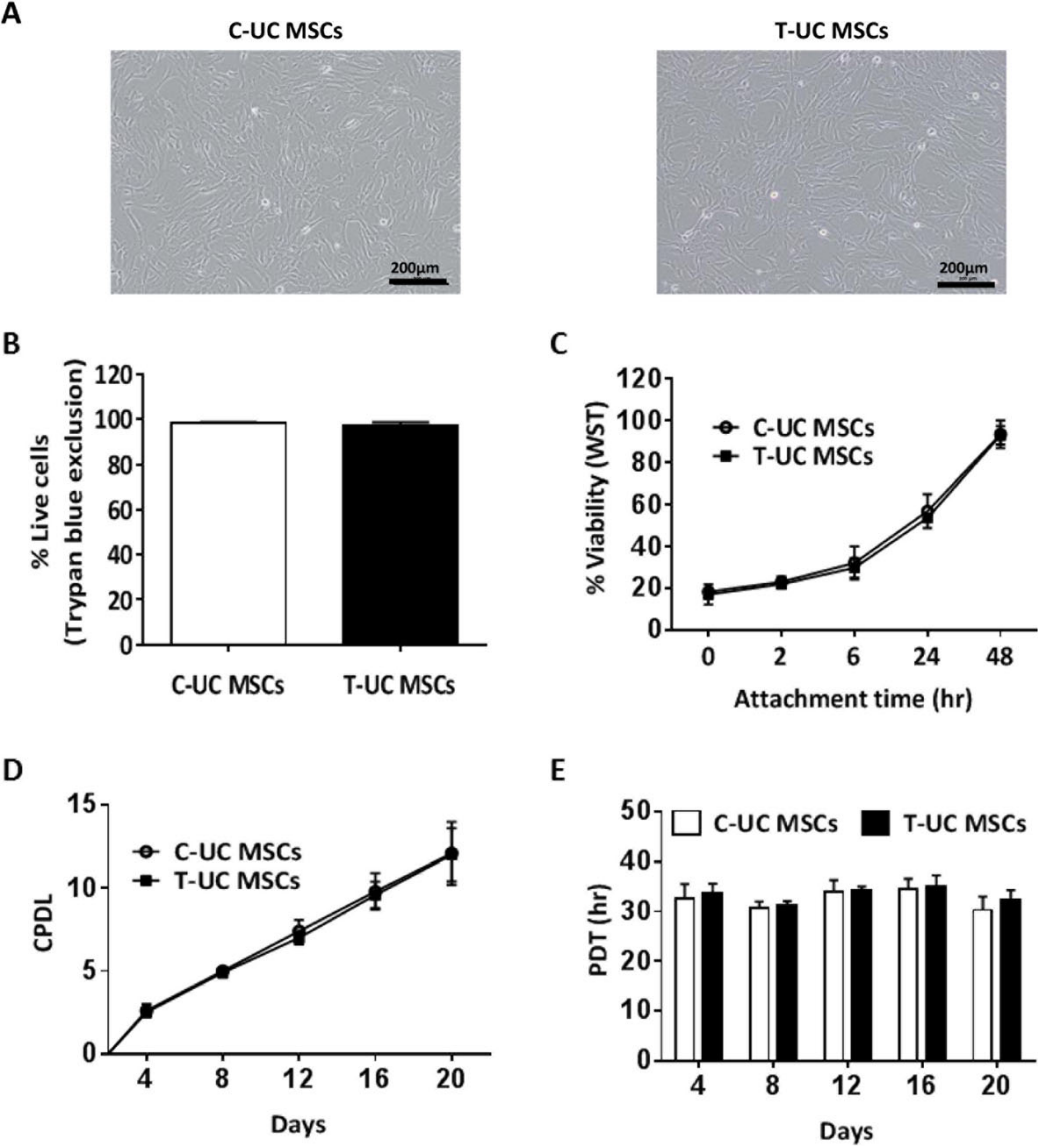
Characterization of C- and T-UC MSCs at passage 10. **a** Fibroblast-like morphology of cells (magnification; × 100). **b** Viability evaluated by trypan blue exclusion. **c** Viability evaluated by water-soluble tetrazolium salt (WST) assay. **d** Calculation of cumulative population-doubling level (CPDL). **e** Population-doubling time (PDT). Bar charts represent mean ± standard deviation; statistically significant at *p* < 0.050

Both C- and T-UC MSCs showed fibroblast-like morphology (Fig. 1a). Viabilities of C- and T-UC MSCs were 98.25 ± 0.50% and 97.25 ± 1.50%, respectively, in trypan blue assay (Fig. 1b). In WST results, the viabilities were 32.1 ± 7.95 in C-UC MSCs and 29.8 ± 4.76 in T-UC MSCs at 6 h and 93.6 ± 6.68 in C-UC MSCs and 93.1 ± 4.48 in T-UC MSCs at 48 h (Fig. 1c). There was no significant difference between C- and T-UC MSCs.

Results of the proliferative capacity showed that the values of CPDL in C- and T-UC MSCs were 12.1 ± 1.9 and 12.0 ± 1.6, respectively, by day 20 (Fig. 1d). The mean values of PDT in C- and T-UC MSCs were 32.36 ± 1.86 h and 33.36 ± 1.58 h, respectively (Fig. 1e). No significant difference in proliferative capacity was detected between C- and T-UC MSCs.

### Macroscopic evaluation

**Fig. 2:**
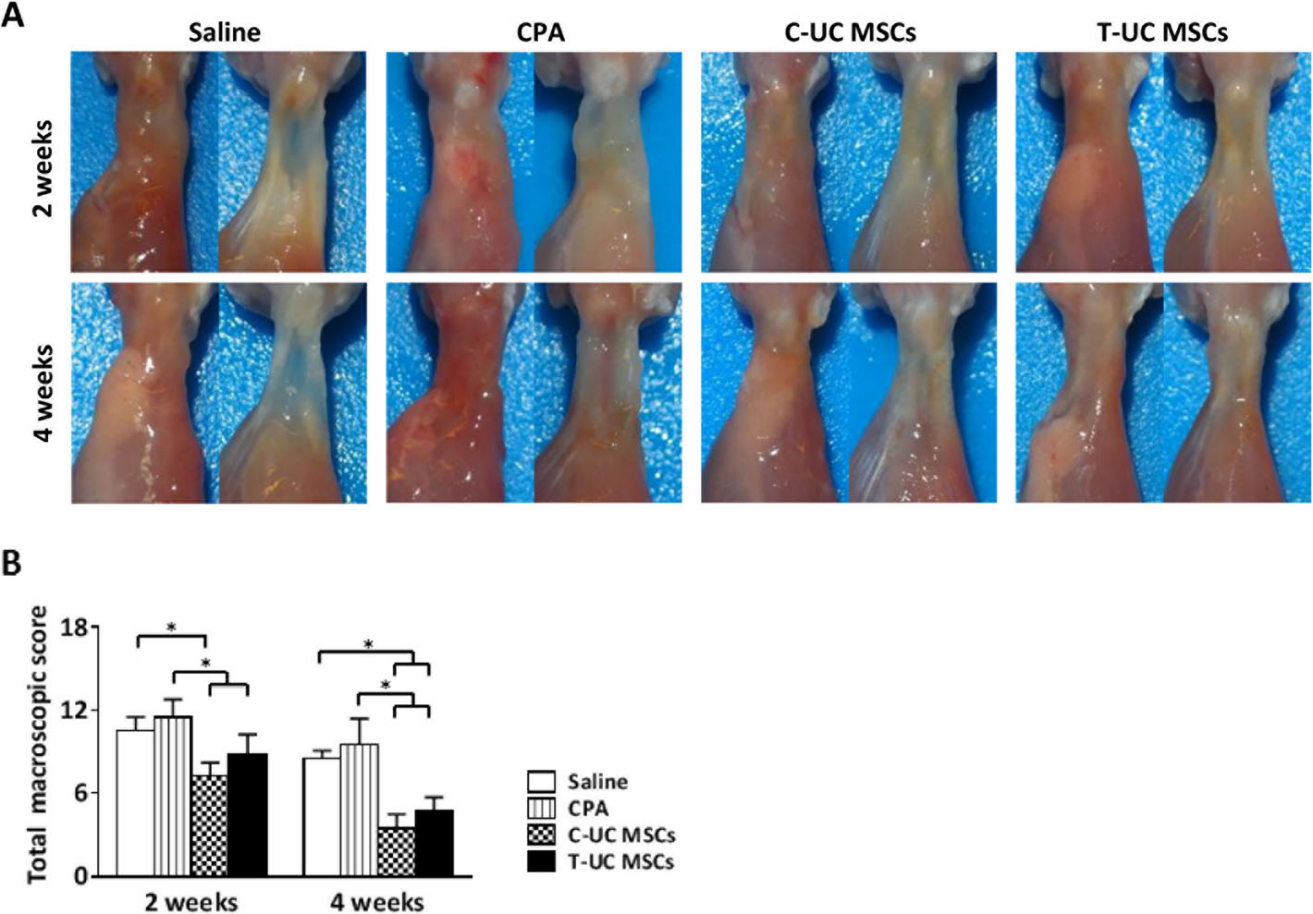
Macroscopic evaluation of regenerated tendons at 2 and 4 weeks after injection with saline, CPA, and C- and T-UC MSCs. **a** Macroscopic appearance of the supraspinatus tendon (left side: shape of a supraspinatus tendon immediately after detachment; right side: shape of the tendon without the loose connective tissue surrounding the defect site to observe the original defect). **b** The total macroscopic score. Bar charts represent mean ± standard deviation; statistically significant at *p* < 0.050

The total macroscopic score was significantly lower in the T-UC MSCs (8.75 ± 1.50) than in the CPA group (11.50 ± 1.29) (*p* = 0.044) at 2 weeks. Especially, the scores of neighboring tendon and the defect level were at least 0.5 points less in the T-UC MSCs than in the CPA group. No significant difference was found between C-UC MSCs (7.25 ± 0.96) and T-UC MSCs at 2 weeks. At 4 weeks, the total macroscopic score was significantly lower in the T-UC MSCs (4.75 ± 0.96) than in the CPA group (9.50 ± 1.91) (*p* = 0.001). The scores of inflammation, surface, defect size, neighboring tendon, defect level, swelling/redness, and the connecting surrounding tissue and slidability were at least 0.5 points lower in the T-UC MSCs than in the CPA group. There was no significant difference between the C-UC MSCs (3.50 ± 1.00) and the T-UC MSCs at 4 weeks (Fig. 2a, b).

### Histological evaluation

**Fig. 3:**
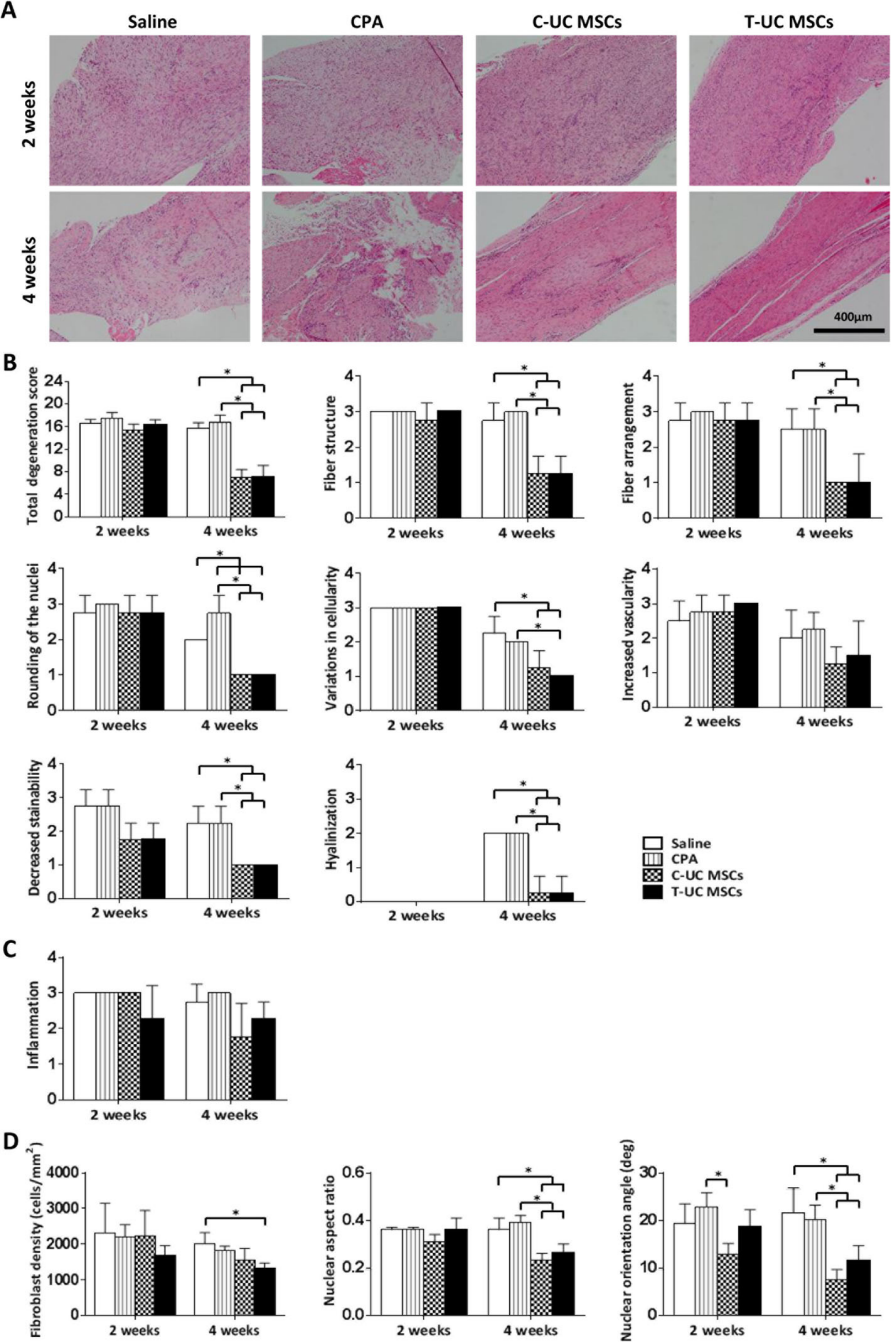
Histological evaluation of regenerated tendon at 2 and 4 weeks after injection with saline, CPA, and C- and T-UC MSCs. **a** H&E staining of tendon (magnification; × 100). **b** The total degeneration score and detailed parameters. **c** Inflammation at the tendon defect. **d** Density, nuclear aspect ratio, and nuclear orientation angle of fibroblasts in the tendon. Bar charts represent mean ± standard deviation; statistically significant at *p* < 0.050

The total degeneration score showed no significant difference between T-UC MSCs and CPA groups at 2 weeks. After 4 weeks, the total degeneration score was significantly reduced in the T-UC MSCs group, 7.00 ± 2.16, compared with the CPA group, 17.25 ± 0.96 (All *p* < 0.001). The scores of fiber structure, fiber arrangement, rounding of nuclei, variations in cellularity, decreased stainability, and hyalinization were significantly lower in the T-UC MSCs groups than in the CPA groups. There was no significant difference in total degeneration score between C-UC MSCs (7.00 ± 1.41) and T-UC MSCs at 4 weeks. In vascularity, there were no significant differences among groups (Fig. 3a, b).

There was no significant immune response in the MSCs, and the inflammation scores were reduced in the C- and T-UC MSCs groups compared to the saline and CPA groups at 4 weeks, but there were no significant differences among groups at both 2 and 4 weeks (Fig. 3a, c).

At 2 weeks, the values of fibroblast density, nuclear aspect ratio, and nuclear orientation angle were not significantly different between T-UC MSCs and CPA groups. After 4 weeks, the fibroblast density was lower in T-UC MSCs (1308.32 ± 164.69 cells/mm^2^) than in the CPA group (1820.98 ± 117.20 cells/mm^2^) (*p* = 0.009), but there was no significant difference. The nuclear aspect ratio was significantly decreased in T-UC MSCs (0.26 ± 0.04) compared with the CPA group (0.39 ± 0.03) (*p* = 0.003). There was no significant difference between the C-UC MSCs (0.23 ± 0.03) and T-UC MSCs group at 4 weeks. The nuclear orientation angle was also lower in the T-UC MSCs (11.49 ± 3.15) than in the CPA group (20.07 ± 3.15) (*p* = 0.032). There was no significant difference between C-UC MSCs (7.59 ± 2.07) and T-UC MSCs at 4 weeks (Fig. 3a, d). Heterotopic ossification was not observed in any group at any time point (Fig. 3a).

**Fig. 4:**
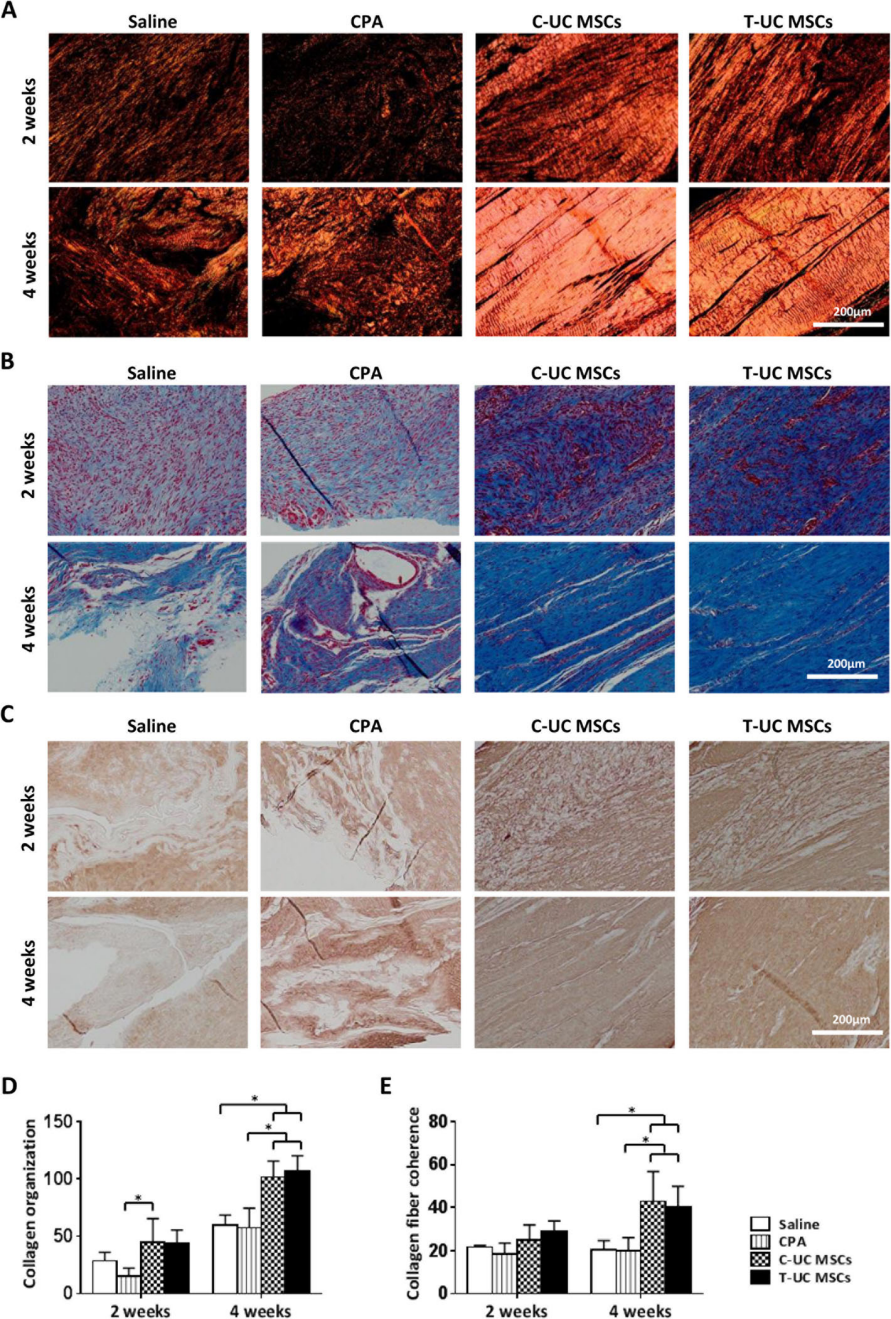
Quantification of collagen matrix changes in regenerated tendon at 2 and 4 weeks after injection with saline, CPA, and C- and T- UC MSCs. **a** PSR staining of the tendon (magnification; × 200). **b** MT staining of the tendon (magnification; × 200). **c** IHC for type I collagen of the tendon (magnification; × 200). **d** Collagen organization in the tendon. **e** Collagen fiber coherence of the tendon. Bar charts represent mean ± standard deviation; statistically significant at *p* < 0.050

The scores of collagen organization and collagen fiber coherence were not significantly different between T-UC MSCs and CPA groups at 2 weeks. After 4 weeks, collagen organization increased significantly in the T-UC MSC group (106.83 ± 13.46) compared with the CPA group (57.46 ± 16.94) (*p* = 0.002). There was no significant difference between C-UC MSCs (101.78 ± 13.89) and T-UC MSC groups at 4 weeks (Fig. 4a, d). The collagen fiber coherence was also higher in the T-UC MSC groups (39.94 ± 9.95) than in the control groups (20.07 ± 5.99) (*p* = 0.008). No significant differences were found between C-UC MSCs (42.95 ± 13.89) and T-UC MSCs at 4 weeks (Fig. 4a, e).

Both collagen deposit and type I collagen formation were higher in the T-UC MSCs group than those in the CPA group at 2 weeks. At 2 weeks, the CPA group showed low collagen formation which consists of low-density type I collagen, and it remained until 4 weeks. However, the T-UC MSCs group showed higher collagen formation which consists of high-density type I collagen than that in the CPA group, and the type I collagen in T-UC MSCs group became more dense at 4 weeks. Moreover, there was no significant difference between C-UC MSCs and T-UC MSCs groups at 2 and 4 weeks (Fig. 4b, c).

**Fig. 5:**
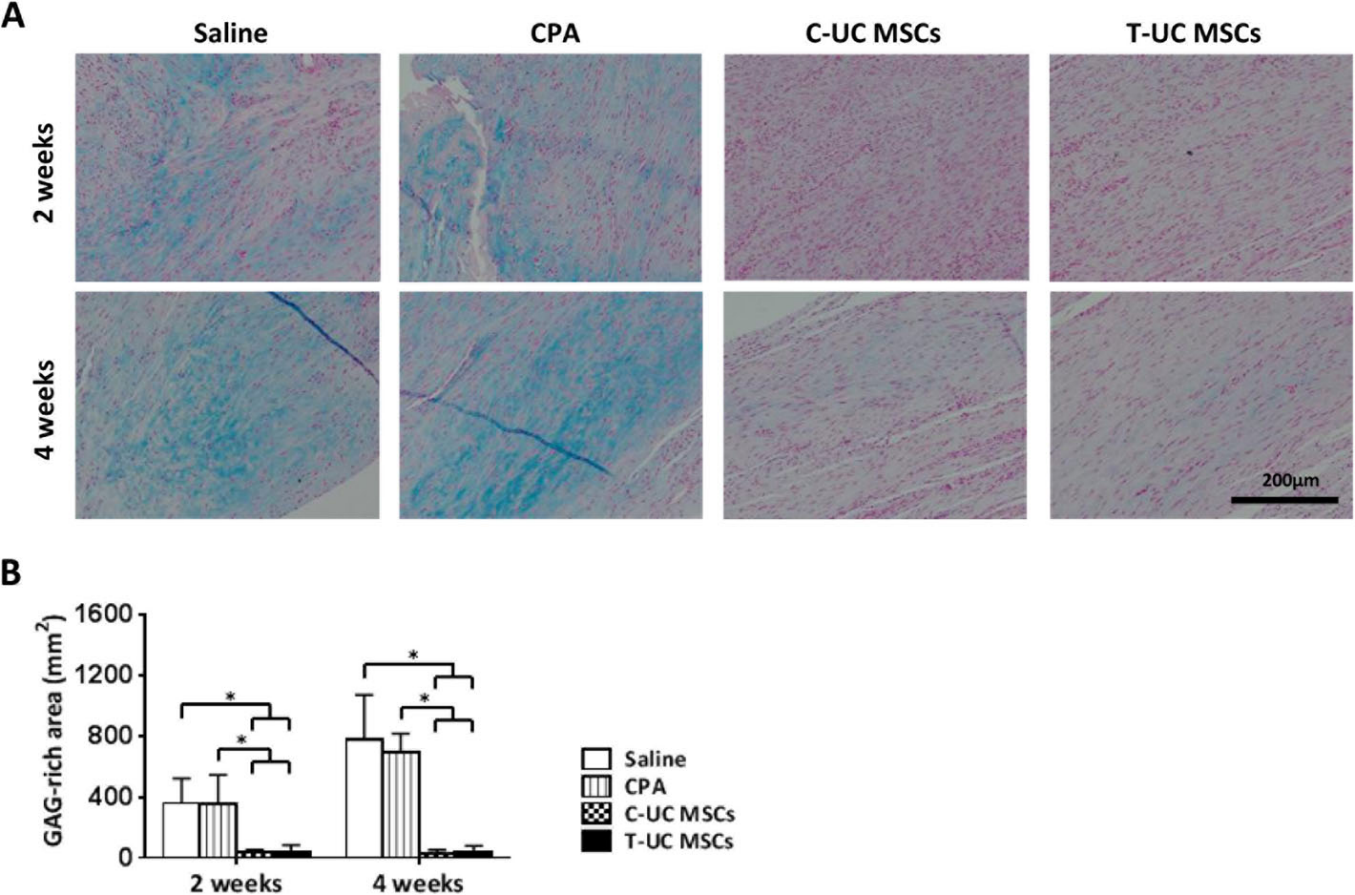
Quantification of GAG-rich area in the regenerated tendons at 2 and 4 weeks after injection with saline, CPA, and C- and T-UC MSCs. **a** Alcian blue staining of the tendon (magnification; × 200). **b** GAG-rich area of the tendon. Bar charts represent mean ± standard deviation; statistically significant at *p* < 0.050

In terms of GAG-rich area, the area was significantly smaller in the T-UC MSCs (42.49 ± 36.59 N) than in the CPA group (358.05 ± 187.26 N) at 2 weeks (*p* = 0.001), and there was no significant difference between C-UC MSCs (40.21 ± 13.99 mm^2^) and T-UC MSCs. After 4 weeks, the GAG-rich area was less in the T-UC MSC groups (36.59 ± 44.21 mm^2^) compared with that of the CPA group (690.99 ± 125.45 mm^2^) (*p* = 0.024). There was no significant difference between C-UC MSCs (30.72 ± 21.79 mm^2^) and T-UC MSCs (Fig. 5a, b).

### Biomechanical evaluation

**Fig. 6:**
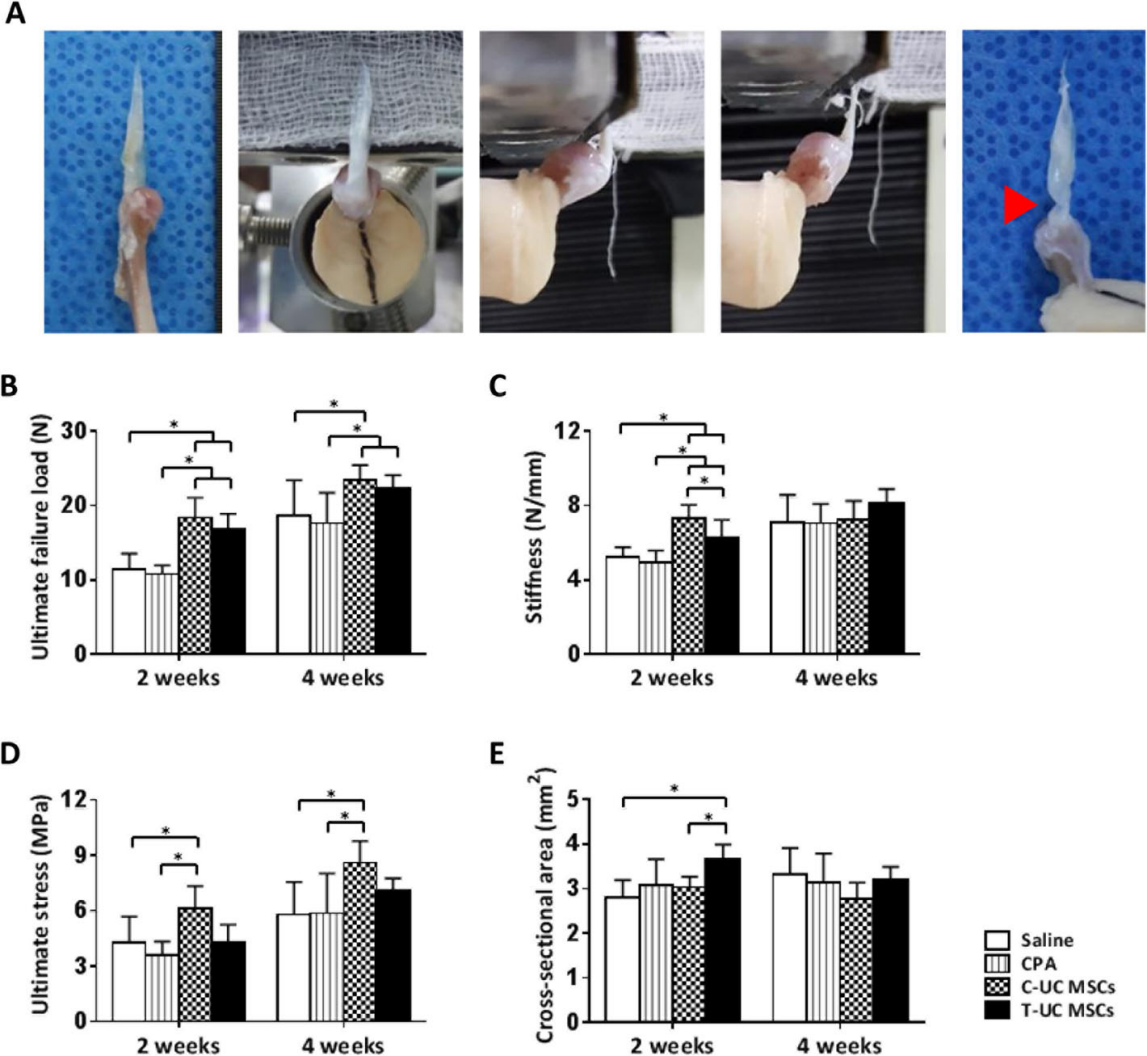
Biomechanical procedure and quantification of the properties of regenerated tendons at 2 and 4 weeks after injection with saline, CPA, and C- and T-UC MSCs. **a** A harvested supraspinatus tendon attached to the proximal humerus, and the procedure outline the biomechanical experiment. **b** Ultimate failure load. **c** Stiffness. **d** Ultimate stress. **e** Cross-sectional area of the supraspinatus tendon at defect site. Bar charts represent mean ± standard deviation; statistically significant at *p* < 0.050

The value of ultimate failure load was significantly higher in the T-UC MSCs (16.69 ± 2.20 N) than in the CPA group (10.79 ± 1.18 N) at 2 weeks (*p* < 0.001). After 4 weeks, the value was also enhanced in the T-UC MSCs (22.33 ± 1.75 N) compared with the control groups (17.59 ± 4.12 N) (*p* = 0.043). There was no significant difference between C-UC MSCs at 2 weeks (18.40 ± 2.67 N) and at 4 weeks (23.40 ± 2.06 N) and T-UC MSCs at both 2 and 4 weeks (Fig. 6b).

The value of stiffness was improved significantly in the T-UC MSCs group (6.27 ± 0.95 N/mm) compared with that of the CPA group (4.97 ± 0.61 N/mm) at 2 weeks (*p* = 0.008). There was no significant difference between C-UC MSCs (7.30 ± 0.74 N/mm) and T-UC MSCs groups. After 4 weeks, stiffness was higher in T-UC MSCs (8.08 ± 0.81 N/mm) than that in CPA group (7.04 ± 1.04 N/mm), but there was no significant difference between groups (Fig. 6c).

In ultimate stress, there was no significant difference between T-UC MSCs and CPA groups at 2 weeks due to the increased cross-sectional area of tendon in T-UC MSCs. However, after 4 weeks, ultimate stress was higher in T-UC MSCs (7.03 ± 0.71 MPa) than that in CPA group (5.85 ± 2.16 MPa) although there was no significant difference (Fig. 6d, e).

### UC MSCs trafficking

**Fig. 7:**
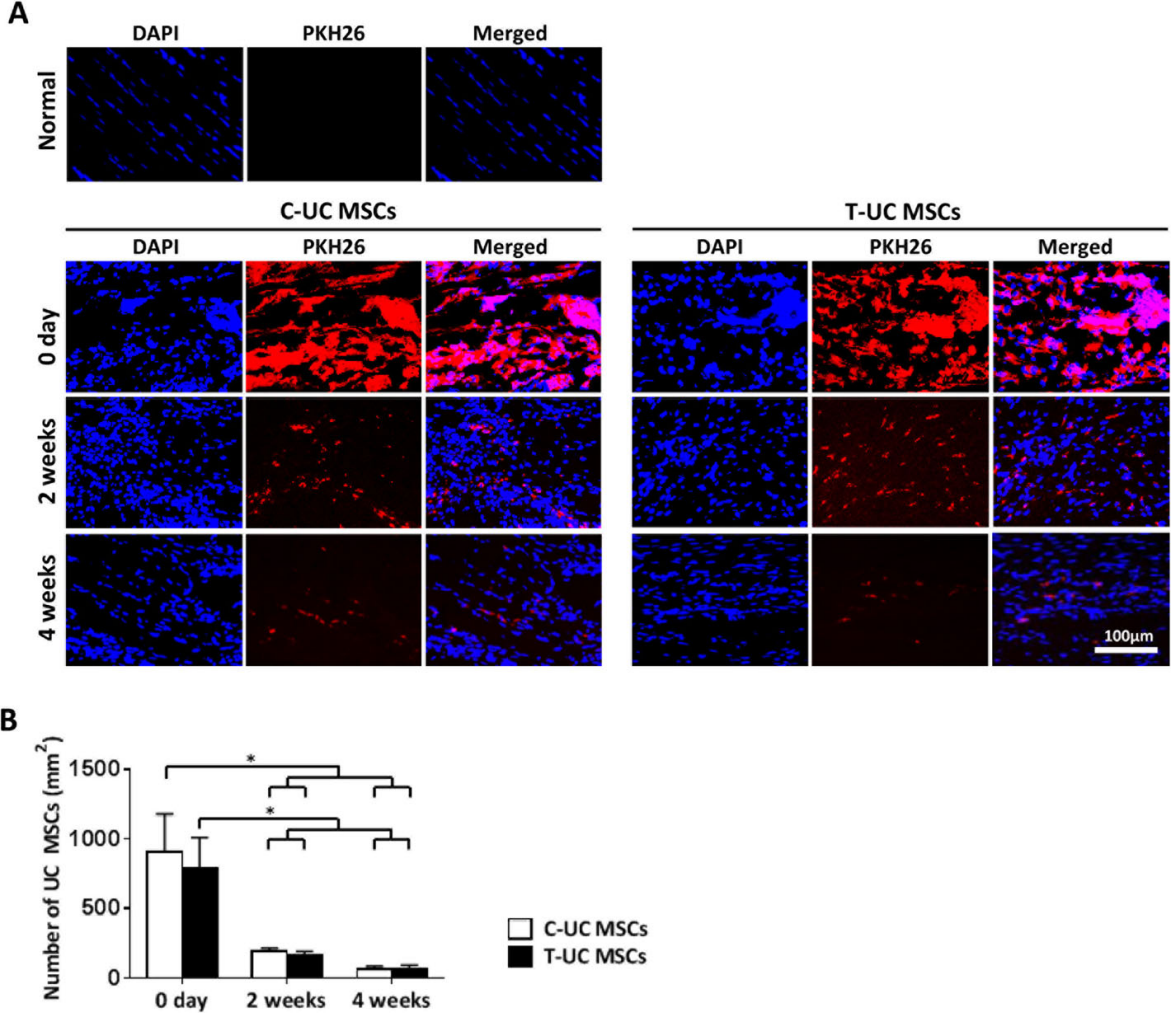
Cell trafficking for UC MSCs within the tendon and quantification of the UC MSCs on the day after injection and at 2 and 4 weeks after injection. **a**: PKH26-labeled UC MSCs with DAPI within the tendon (magnification; × 400). **b** The number of the UC MSCs per area (mm^2^). Bar charts represent mean ± standard deviation; statistically significant at *p* < 0.050

At 2 weeks, the mean number of PKH26-labeled cells per area was reduced by 21.40% in T-UC MSCs compared to that at 0 day (*p* < 0.000), and there was no significant difference between C-UC MSCs (194.15 ± 24.28 cells/mm^2^) and T-UC MSCs (168.83 ± 24.11 cells/mm^2^). After 4 weeks, the mean number of cells significantly decreased 8.77% in T-UC MSCs compared to that at 0 day, and there was no significant difference between C-UC MSCs (64.07 ± 22.43 cells/mm^2^) and T-UC MSCs (69.17 ± 27.07 cells/mm^2^) (*p* < 0.000) (Fig. 7).

## Discussion

The most important findings of this study include (1) The T-UC MSCs exhibited fibroblast-like morphology and above 97% viability and stable proliferation comparable to that of the C-UC MSCs at passage 10. (2) In a rat model of FTD, macroscopic appearance improved in T-UC MSCs compared with the control (CPA) group at both 2 and 4 weeks in terms of inflammation, defect size, neighboring tendon, swelling/redness, and the connecting surrounding tissue and slidability, and there was no significant difference between T- and C-UC MSCs groups. (3) Histologically, compared to the control group, the nuclear aspect ratio, orientation angle of fibroblasts, collagen organization, and fiber coherence were improved by 33.33%, 42.75%, 1.86-fold, and 1.99-fold and GAG-rich area decreased by improved by 88.13% at 2 weeks and 94.70% at 4 weeks. All improved values of T-UC MSCs group were comparable to those of C-UC MSCs group. (4) The ultimate failure load was 1.55- and 1.25-fold higher in the T-UC MSCs than in the control group at both 2 and 4 weeks, respectively, and there was no significant difference between C- and T-UC MSCs. Taken together, these results showed that T-UC MSCs showed comparable survival and proliferation to those of C-UC MSCs, and treatment with T-UC MSCs improved the regeneration of rotator cuff tendon in terms of macroscopic, histological, and biomechanical properties, which were comparable to those of treatment with C-UC MSCs.

MSCs are known to be effective in preclinical immunomodulatory and regenerative studies; however, the use of MSCs in clinical practice is a concern in that freshly thawed MSCs may degrade during the thawing procedure. Clinical trials generally use freshly thawed cells that are readily retrieved from cryostorage whereas preclinical experiments use fresh cells [34]. Thus, the difference in effectiveness between freshly thawed cells and fresh cells must be demonstrated. Although thawing afresh minimally affected cell growth, differentiation, and general phenotypes, viability and bioactivity were affected [16]. Some studies reported that continuously cultured cells exhibit ~ 100% viability after harvest afresh, whereas freshly thawed cells have only ~ 70% viability [18] and in worst cases, ~ 50% of viability [35]. Conversely, T-UC MSCs, in this study, showed above 97% viability of cells with comparable morphology and proliferation compared with the C-UC MSCs even after fresh thawing procedure. Cell viability depends on the thawing method, duration of cold storage, and reagents used [36]. Relatively short storage periods [37] and optimal concentration of DMSO (10%) are crucial factors contributing to high cell viability in cryopreserved conditions [35, 37–39]. Other conditions not mentioned here may have an impact on the high cell viability in this study. In conclusion, our results suggest successful cryopreservation of UC MSCs to maintain cell conditions similar to continuously cultured MSCs after fresh thawing procedure [37].

Recent studies reported the efficacy of cryopreserved MSCs with inconsistent results in animal models. Moll et al. demonstrated that freshly thawed MSCs showed reduced viability and increased apoptosis and were associated with negative immunomodulatory effects and blood regulation resulting in faster complement-mediated elimination after blood exposure [16]. However, Cruz et al. reported that freshly thawed human BM MSCs could regulate allergic airway inflammation in an immunocompetent mouse model, and no significant differences were found between cultured MSCs and freshly thawed MSCs [18]. In fact, the freshly thawed MSCs numbered 1.3 × 10^6^ cells, whereas the cultured MSCs were only 1 × 10^6^ cells considering dead cells because the freshly thawed MSCs showed a viability of 70% [18]. These results suggested that an adequate number of living cells are important for healing capacity, and thus, the high cell viability of freshly thawed MSCs is also a crucial factor determining the efficacy comparable to the fresh cultured MSCs. In this study, we used the same number of MSCs in both C- and T-UC MSCs for animal experiments and found comparable potential for tendon regeneration using T-UC MSCs and C-UC MSCs. This study demonstrated that the high viability of cells and adequate cell numbers positively contributed to recovery from tendon injury.

European studies of ex vivo-expanded cells generally use passages 1–4, whereas passages beyond 5 were commonly used in company-sponsored phase three trials [34]. In patients with acute graft-versus-host disease, 75% of early-passage BM MSCs (passage 1–2) survived after 1 year and only 21% of later passage MSCs (passages 3–4) survived [40], suggesting the reduced therapeutic value and potency of high passage MSCs [16]. By contrast, we found the efficacy of UC MSCs even though passage 10 was used (although not compared by passage) because UC MSCs remain immature even after several passages, and no genetic changes were detected following long-term expansion [41]. Further, using our isolation and culture method to manipulate UC MSCs, no difference was found in CPDL, proliferation, pluripotent stem cell markers, or differentiation among UCMSCs with passages 2, 3, 7, and 9, and the stemness persisted even with passaging and senescence. Telomeric results showed no difference according to the passage [19]. Furthermore, Zhuang et al. reported that human UC MSCs after passage 15 exhibited stronger immunosuppressive activity than those after early passage (passage 3), suggesting that at later passages, human UC MSCs represent a good therapeutic option for patients with graft versus host disease and other immune diseases [42]. Hence, it is suggested that MSCs at an early passage might not necessarily be better, and T-UC MSCs at passage 10 exhibit enough positive effect on tendon regeneration suggesting the possibility of using UC MSCs as “off-the-shelf” treatments for patients clinically.

Although there were histological and biomechanical improvements until 4 weeks after UC MSCs injection, most of the injected UC MSCs faded away at the injured tendon over time. Recently, several studies have shown that the healing effect of MSCs comes from the paracrine effect, which secrete cytokines and growth factors to recruit, proliferate, and differentiate tissue-specific progenitor cells to synthesis-specific matrix [43]. Moreover, MSCs have the ability to modulate local inflammatory environment by regulating inflammatory cell function by inducing macrophage recruitment and polarization to alternatively activated macrophage suppressing inflammation and the inflammatory-related cells [44, 45]. Thus, the injected UC MSCs disappeared over time, but the local environment influenced by UC MSCs has a positive effect on the regeneration of the structure of the injured tendon. However, more study is needed to explain the healing effects of UC MSC on tendon regeneration.

There are several limitations in this study. First, we did not perform gene expression and protein synthesis evaluation, and it is difficult to prove the mechanism of the healing effects of UC MSCs. Second, we only investigated tendon regeneration at 2 and 4 weeks after injecting UC MSCs, which was a brief period to adequately determine the efficacy of T-UC MSCs. Thus, a long-term follow-up study is needed to confirm the sustained efficacy of treatment with C- and T-UC MSCs.

## Conclusions

The morphology, viability, and proliferation of T-UC MSCs were comparable to those of C-UC MSCs. Treatment with T-UC MSCs could induce tendon regeneration of FTD at the macroscopic, histological, and biomechanical levels comparable to treatment with C-UC MSCs.

## Supplementary information

**Additional file 1 Procedure of experiments.**

**Additional file 2 Modified macroscopic evaluation system.**

**Additional file 3.**

**Additional file 4 UC MSCs Trafficking.**

## Data Availability

The datasets used and/or analyzed during the current study are available from the corresponding author on reasonable request.

## Abbreviations

MSCs: Mesenchymal stem cells
FTD: Full-thickness tendon defect
CPA: Cryopreserved agent
C-UC MSCs: Cultured umbilical cord-derived MSCs
T-UC MSCs: Freshly thawed umbilical cord-derived MSCs
BM MSCs: Bone marrow-derived MSCs
AD MSCs: Adipose tissue-derived MSCs
UC MSCs: Umbilical cord-derived MSCs
WST: Water-soluble tetrazolium salt
OD: Optical density
CPDL: Cumulative population-doubling level
PDT: Population-doubling time
SST: Supraspinatus tendon tissue
EDTA: Ethylendiaminetetracetic acid
H&E: Hematoxylin and eosin
ROI: Regions of interest
PSR: Picrosirius red
Saf-O: Safranin-O/fast green
GAG: Glycosaminoglycan
ANOVA: One-way analysis of variance
