# Supplementary figures and images for: Regeneration of a full-thickness defect of rotator cuff tendon with freshly thawed umbilical cord-derived mesenchymal stem cells in a rat model
Source: Stem Cell Res Ther. 2020 Sep 7;11:387. doi: 10.1186/s13287-020-01906-1 (PMC7487485; doi:10.1186/s13287-020-01906-1)

**Additional File 1**

**
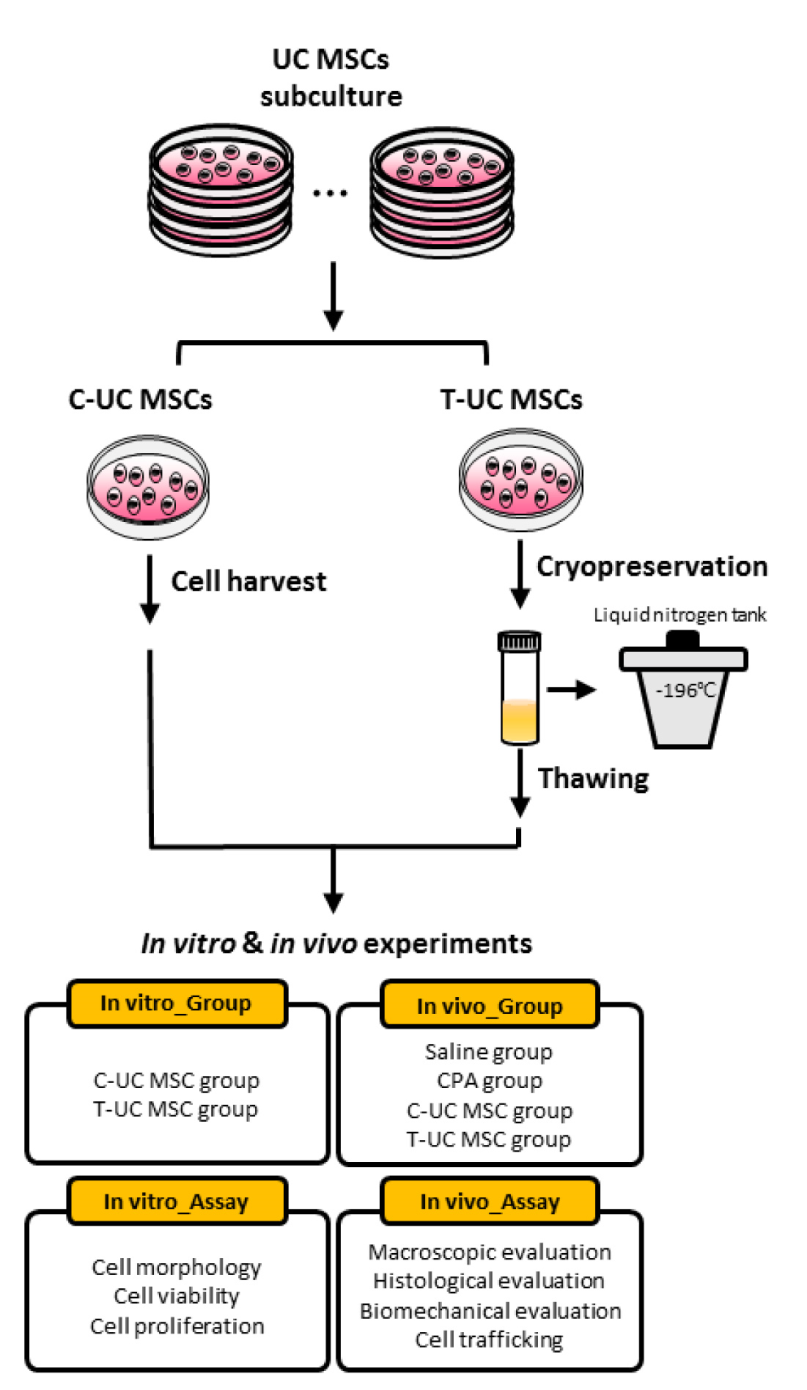
Additional file 1. Procedure of experiments.**

Supplement: Supplementary file 1 — Additional file 1 Procedure of experiments. [file 13287_2020_1906_MOESM1_ESM.docx]
