# Supplementary material for: Regeneration of a full-thickness defect of rotator cuff tendon with freshly thawed umbilical cord-derived mesenchymal stem cells in a rat model
Source: Stem Cell Res Ther. 2020 Sep 7;11:387. doi: 10.1186/s13287-020-01906-1 (PMC7487485; doi:10.1186/s13287-020-01906-1)
Supplement: Supplementary file 2 — Additional file 2 Modified macroscopic evaluation system. [file 13287_2020_1906_MOESM2_ESM.docx]

**Additional File 2**

**Additional file 2. Modified macroscopic evaluation system** (1)

| **Parameters** | **Score** |
| --- | --- |
| **Tendon rupture** |  |
| Non existing | 0 |
| Existing | 1 |
| **Inflammation** |  |
| Non existing | 0 |
| Existing (edema, swelling, redness) | 1 |
| **Tendon surface** |  |
| Intact, smooth | 0 |
| Uneven, harsh | 1 |
| **Neighbouring tendon** |  |
| Unchanged | 0 |
| Changed (colour, thickness, surface) | 1 |
| **Level of the defect** |  |
| At the niveau of tendon surface | 0 |
| Prominent above the level of tendon | 1 |
| **Defect size** |  |
| Size about 3 mm | 0 |
| Augmented or non delimitable | 1 |
| **Swelling/redness of tendon** |  |
| No swelling/no redness | 0 |
| Palpable swelling, no redness | 1 |
| Palpable swelling with redness | 2 |
|  |  |
| **Connection surrounding tissue and slidability** |  |
| No adnated, slidable | 0 |
| Adhesion, not slidable | 1 |
| **Tendon thickness (Shape of tendon)** |  |
| Normal | 0 |
| Slightly thickened (~15%) | 1 |
| Moderately thickened (15~30%) | 2 |
| Intensely thickened (30%~) | 3 |
| **Color of tendon** |  |
| Bright white | 0 |
| Translucent, dull white, rose | 1 |
| **Single strains of muscle** |  |
| Normal conjoined | 0 |
| Adhesion, heavily adnated | 1 |
| **Transition of the construct to the surrounding healthy tissue** |  |
| No transition detectable | 0 |
| Gaps, callus, cracks in the transition area | 1 |
| **Total** | **15** |

1. Stoll C, John T, Conrad C, Lohan A, Hondke S, Ertel W, et al. Healing parameters in a rabbit partial tendon defect following tenocyte/biomaterial implantation. Biomaterials. 2011;32(21):4806-15.
