# Supplementary material for: Regeneration of a full-thickness defect of rotator cuff tendon with freshly thawed umbilical cord-derived mesenchymal stem cells in a rat model
Source: Stem Cell Res Ther. 2020 Sep 7;11:387. doi: 10.1186/s13287-020-01906-1 (PMC7487485; doi:10.1186/s13287-020-01906-1)
Supplement: Supplementary file 3 — Additional file 3. [file 13287_2020_1906_MOESM3_ESM.docx]

**Additional File 3**

**Additional file 3. Immunohistochemistry**

Randomly selected slides were used for antigen retrieval (Proteinase K, S3020, Dako UK Ltd., Ely, UK) and were blocked with Peroxide Block solution (ACA125, ScyTek, Logan, UT, USA) and Super Block solution (AAA500, ScyTek). After blocking, the slides were incubated with a rabbit antibody to type 1 collagen (1:300 dilution, ab34710, Abcam Cambridge, UK) for overnight at 4℃. Primary antibody was detected with EnVisionTM+ Horse Radish Peroxidase (HRP) Systems (DAKO Corporation, Hamburg, Germany) for 30 minutes and incubated in DAB+ enzyme (premised 3,3′ diaminobenzidine (DAB) plus) (C09-12, GBI Labs, Mukilteo, WA, USA) for 1 min. All IHC staining for type 1 was carried out at the same time to reduce variables during the procedure.
