# Supplementary material for: Regeneration of a full-thickness defect of rotator cuff tendon with freshly thawed umbilical cord-derived mesenchymal stem cells in a rat model
Source: Stem Cell Res Ther. 2020 Sep 7;11:387. doi: 10.1186/s13287-020-01906-1 (PMC7487485; doi:10.1186/s13287-020-01906-1)
Supplement: Supplementary file 4 — Additional file 4 UC MSCs Trafficking. [file 13287_2020_1906_MOESM4_ESM.docx]

**Additional File 4**

**Additional file 4. UC MSCs Trafficking**

When cells reached 60% to 80% confluence, they were detached by incubation for 5 minutes with 0.25% trypsin EDTA. The suspension was centrifuged at 400xg for 5 min and washed three times with PBS. After aspirating the supernatant in UC MSC containing tubes, the cells were resuspended gently in 1mL of the dilution buffer and mixed with an equal volume of the labeling solution containing 4x10^-2^_M_ PKH26 in the dilution buffer. Then, the cells were incubated at 5 min at room temperature and the reaction was terminated by addition 2 mL of FBS. The suspension was centrifuged at 400xg for 5 min and the cells were washed three times with PBS. After labeling, the cells were counted by hemocytometer, and confirmed by fluorescence microscope (Leica DMI 4000B, Leica, Wetzlar, Germany) before injection.

The tissue was harvested immediately after injection and two and four weeks after injection, and used for evaluation. We harvested only supraspinatus tendon and the harvested tissues were immediately fixed in 4% (w/v) PFA for 24 hours. The tissues were treated with consecutive 10%, 15% and 20% sucrose/PBS solutions at 4℃ for 12h respectively. The tissues were embedded in O.C.T. compound (Tissue-Tek, Miles, USA) with subsequent freezing of the block at -80℃. To evaluate the survival ability of the PKH 25 labeled UC MSCs, the specimens were sectioned (7 μm) with a freezing microtome (Leica CM3050S, Leica, Wetzlar, Germany) and carefully trimmed until we found middle side of tendon. The slides were mounted with Vectashield containing DAPI (Vector Laboratories Inc., Burlingame, CA). Five fields were randomly selected in the slide and high-powered images (x400) were obtained by fluorescence microscopy. The PKH26 positive cells coincident with 4′,6-diamidino-2-phenylindole (DAPI) were counted per area and the mean number were recorded by image J (1)

1. Kang ES, Ha KY, Kim YH. Fate of transplanted bone marrow derived mesenchymal stem cells following spinal cord injury in rats by transplantation routes. J Korean Med Sci. 2012;27(6):586-93.
